# Therapeutic Notes

**Published:** 1907-09-21

**Authors:** 


### THERAPEUTIC NOTES.

### The Rise in Value of Morphine and Codeine.

At the end of June morphine hydrochloride was quoted 6s. 3d. per ounce, but by a steady series of advances the price has been raised to 9s. 9d. At the beginning of the year, when many hospital contracts were probably entered upon, the quotation was 5s. 3d., so that obviously contractors who neglected to cover themselves are suffering very considerable loss. The price of codeine has similarly advanced; in January of this year this alkaloid could have been bought in contract quantities at about 10s. per ounce; by the end of June the price was 11s. 6d., while to-day it is 16s. per ounce. The cause in both instances is the rise in value of the raw material from which the alkaloids are extracted, and the position of the opium market is becoming more difficult every day. Owing to the unfavourable weather that has prevailed in the growing districts in Turkey the poppy crops have suffered very severely, and it is estimated that the outturn during the present season will fall at least 1,000 cases below the world's requirements. The question arises as to how far the extreme prices quoted for codeine and morphine may possibly interfere with their use. In the case of many drugs high values tend to check consumption and to encourage the use of suitable substitutes, but in the case of morphine and codeine it is not probable that general resort will be had to narcotics of a less expensive character. It is impossible to foresee what the trend of the market in these two products will be in the immediate future. Preparations of opium such as the tincture and the extract have, of course, advanced in sympathy with the value of the raw material.

### The Chloroforms of Aconite and Belladonna.

The methods which, up to the present, have been adopted for the preparation of these two products have been unsatisfactory, and according to R. Wright the best method is that which is to be included in the "British Pharmaceutical Codex." It consists of treating the roots of aconite or belladonna—whichever preparation is required—with ammonia, and percolating with a mixture of one of absolute alcohol to seven of chloroform. By this means practically the whole of the alkaloids are taken out of the roots, and a uniform preparation may be obtained.

